# Implementing Artificial Intelligence Algorithms in the Radiology Workflow: Challenges and Considerations

**DOI:** 10.1016/j.mcpdig.2024.100188

**Published:** 2024-12-18

**Authors:** Panagiotis Korfiatis, Timothy L. Kline, Holly M. Meyer, Sana Khalid, Timothy Leiner, Brenna T. Loufek, Daniel Blezek, David E. Vidal, Robert P. Hartman, Lori J. Joppa, Andrew D. Missert, Theodora A. Potretzke, Jerome P. Taubel, Jason A. Tjelta, Matthew R. Callstrom, Eric E. Williamson

**Affiliations:** a Department of Radiology, Mayo Clinic, Rochester, MN; b Mayo Clinic Platform, Mayo Clinic, Rochester, MN; c Center for Digital Health, Mayo Clinic, Rochester, MN; d Department of Information Technology, Mayo Clinic, Rochester, MN

## Abstract

Integration of AI-enabled algorithms into the radiology workflow presents a complex array of challenges that span operational, technical, clinical, and regulatory domains. Successfully overcoming these hurdles requires a multifaceted approach, including strategic planning, educational initiatives, and careful consideration of the practical implications for radiologists' workloads. Institutions must navigate these challenges with a clear understanding of the potential benefits and limitations of both vended and in-house developed AI tools.

Artificial intelligence (AI) is increasingly prevalent in clinical practice, with evidence indicating that it can transform health care. Companies and academic institutions are developing AI algorithms at an unprecedented pace with the aims to improve clinical outcomes, increase care efficiency, reduce costs, enhance the overall patient experience, increase staff satisfaction, address staff shortages, and reduce burnout.^1^

Numerous institutions are developing and investigating AI algorithms under the oversight of an institutional review board. However, to date, few health care institutions as compared with vendors have completed the necessary regulatory controls to receive Food & Drug Administration (FDA) clearance for an AI/machine learning (ML)-enabled medical device.

When referring to AI in radiology, we most often refer to deep learning, a subset of ML-on the basis of the use of neural networks, inspired by biology.^2^ In image processing, convolutional neural networks have reduced the need for extensive feature engineering, therefore reducing the need for human input. Although earlier ML approaches did not meet expectations when rolled out in clinical practice,^3^ recent deep learning algorithms are able to match and even surpass humans in task-specific applications.^4^^,^^5^

At the 2023 Annual Meeting of the Radiologic Society of North America,^6^ more than 90 AI companies were present, compared with just 48 four years previously. Radiology and its digital nature is an appealing area for AI products. Today, nearly 700 FDA cleared or approved AI/ML offerings exist in the market^7^ with 76% (531 out of 691) impacting Radiology.^8^ The following categories are areas in which AI has been identified having potential applications in the radiology practice:

(1) Ordering and scheduling
(2) Intraprocedural guidance
(3) Image acquisition
(4) Image generation
(5) Image analysis
(6) Image interpretation
(7) Patient management and treatment planning
(8) Population health
(9) Departmental analysis and operation

In almost all cases, commercially available AI solutions do not provide answers to complex clinical questions and clinical integration is limited to stand-alone applications, targeting only one calculation or decision. It is therefore complex to integrate a suite of solutions into the clinical workflow.^9^ In addition, several practical and ethical issues exist, limiting the potential for positive impact in the radiology workflow, which requires rethinking the evaluation and integration strategy. For these reasons, institutions with the necessary infrastructure and resources are choosing to leverage their expertise and domain knowledge to develop in-house AI solutions to orchestrate and deploy various AI solutions related to the daily workflow of radiologists.

To enable the successful implementation and integration of AI algorithms in clinical practice and to deliver value to physicians and patients alike, various operational, regulatory, workflow, and technical challenges must be addressed at an institutional level to ensure success.^9^

The objective of this paper is to review the challenges that were identified when our team established a process to enable the discovery and translation of multiple AI algorithms into the clinical workflow. Understanding the challenges can help organizations and research groups decide the best approach for their AI endeavors and set strategic priorities. The challenges are grouped into the following 4 categories: technical, operational, integration, and regulatory.

### Challenges

This section covers the main challenges we have identified. These challenges concern both the translation of AI into the clinical practice with the need for satisfying regulatory requirements and challenges that need to be considered when an institution is onboarding companies that offer radiology AI-based applications.

### Technical

#### Computational Infrastructure

Delivering AI algorithms within clinical settings, whether in simulations or actual workflows subject to FDA regulations, is a complex process heavily reliant on available compute and information technology (IT) resources. Figure 1 outlines a typical architecture for integration, with compute infrastructure tailored to the AI algorithm’s specialized hardware requirements and supported by appropriate IT resources. Turnaround times, often within seconds, necessitate specialized hardware such as edge inference devices, whereas ensuring data security and Health Insurance Portability and Accountability Act adherence is paramount.

Scalable graphical processing unit clusters, storage systems, and cloud computing integration are pivotal for flexible AI deployments. Cloud services offer added scalability and flexibility, balancing on-premises control with the expansive capabilities of cloud computing, whereas network bandwidth upgrades and energy-efficient hardware with advanced cooling solutions optimize resource allocation and management efficiency.

In time-sensitive scenarios such as ultrasound imaging, edge computing plays a vital role by processing data near its source, minimizing latency, and facilitating real-time feedback essential for quick decision-making. Implementing load balancing, distributed computing, and automated management tools further enhances workload distribution, processing times, and system resilience, ensuring optimal performance in AI-enabled clinical environments.

#### Development Environment/Software Tools

To support rapid prototyping and research in AI, essential tools should facilitate data curation, software tracking, experimentation, and collaboration across interdisciplinary teams comprised of IT experts, researchers, clinicians, AI scientists, ML engineers, engineers, and business analysts. Cloud-based solutions and open-access tools offer functionalities for managing large datasets, particularly in multi-modal contexts, with considerations for collaboration and business logic integration. Content management systems integrate image annotation, metadata, and model development capabilities, deployable either locally or in the cloud, although security remains a concern, as open-source research tools may lack thorough vulnerability screening, leading to potential limitations imposed by IT departments.

Furthermore, databases supporting postproduction monitoring are crucial, enabling safety and performance metrics, alongside analytics that track patient outcomes. Platforms for multisite algorithm validation and evaluation that capture radiologist feedback are imperative, along with aligning with best IT practices to mitigate challenges in integration, monitoring, and versioning across discovery and production environments.

#### Clinical System Integration (Technical)

Efficient integration of AI into existing picture archiving and communication system (PACS) and electronic health record (EHR) systems is crucial for seamless clinical workflows, encompassing tasks from exam ordering to delivery of results. This integration necessitates well-documented application programming interfaces conforming to standards,^10^ enabling integration at various levels such as ordering, protocoling, results delivery, and image viewing. However, challenges arise in the openness of clinically used tools for expert modification of AI output, as proprietary file formats may hinder refinement processes, particularly when AI and PACS providers differ.

Shortcomings in PACS tools for correcting AI-generated segmentations pose significant hurdles in production environments, impeding workflow integration. The absence of suitable correction tools complicates adjustments by radiologists and technologists, hindering accuracy assurance and potentially limiting adoption. Moreover, varying operational modes of AI algorithms, whether continuously scanning, triggered by clinician orders, or requiring human input, necessitate parallel systems for simulating clinical workflows, albeit at increased deployment costs.

In large institutions with multiple imaging viewers across departments, standardizing integration becomes even more challenging. Additional hurdles emerge, requiring robust solutions to ensure effective incorporation of AI into diverse clinical settings.

#### Orchestration Engine

Triggering the AI algorithm is one of the challenges that, if not addressed correctly, can negatively affect the clinical utilization of the algorithm. Several available AI solutions rely on the users to trigger the algorithm. This process can be error prone; for example, if the user misidentifies the correct series to route to the algorithm. To address the automation of this process, software solutions have been proposed.^11^ The orchestration engine manages the routing of the data from the imaging modality to the compute engine and finally to the medical image viewer. In many cases, the algorithms depend on a single series; however, in some cases, clinical information and multiple imaging series capturing temporal data is needed. Orchestration engines can be crucial in facilitating seamless workflow and capturing the technical errors and collecting data that can assist in the downstream analysis.

#### Postproduction Monitoring

Postproduction monitoring is integral to the safe and effective deployment of AI applications in clinical settings, ensuring sustained accuracy, effectiveness, and impartiality over time. This requires a robust infrastructure and seamless integration with radiological systems like the PACS viewer, facilitating real-time image analysis and feedback although synchronizing data updates between the AI application and PACS. Key technical aspects include maintaining connectivity and integrating the AI interface within the PACS viewer without disrupting radiologists’ workflows. In addition, a secure data infrastructure supports ongoing learning and model improvement, whereas a performance metric dashboard enables continuous monitoring of application effectiveness and user engagement.

Adherence with health care regulations is imperative to safeguard patient data privacy and security, necessitating tools for regulatory adherence and audit trail maintenance. Embedding feedback mechanisms within clinicians’ workflows and fostering interdisciplinary collaboration platforms encourage ongoing AI refinement, fostering a culture of improvement and learning.

### Operational Challenges

In large institutions with multiple groups working on AI algorithm discovery and development, governance and standardization of the translation process are necessary for a successful AI program. This requires a paradigm shift from the implementation of individual algorithms to designing and building an AI algorithm factory that promotes the implementation of multiple algorithms into the clinical practice, addresses the postproduction needs, and any regulatory requirements. This type of operational framework, covering all phases of the development and integration environment as depicted in Figure 1, will increase efficiency, promote consistency, and support scalability vis-à-vis deployment of multiple algorithms in the practice.

**Figure 1:**
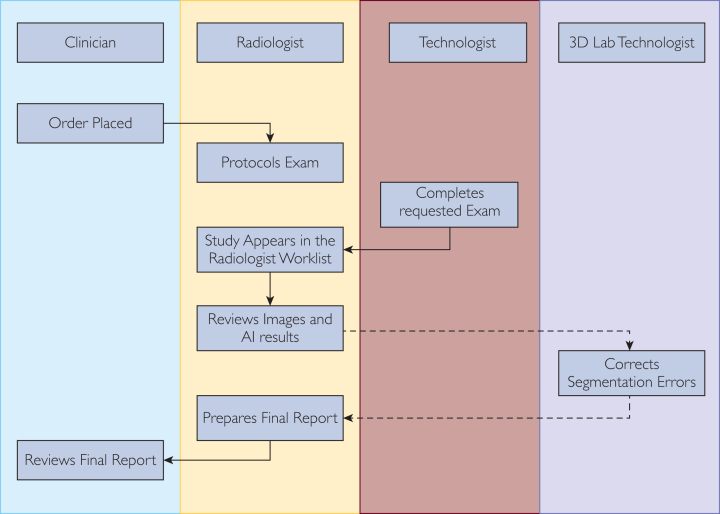
Diagram capturing the complex environment that allows for integration of an AI algorithm into radiology workflows. This figure captures the tools and the interactions that take place when an AI algorithm is integrated in the clinical workflow (PACS, picture archive communication system). AI, artificial intelligence.

In imaging workflows, engaging with clinical radiology division(s) impacted by AI is necessary. It is important, before developing an AI algorithm, to ensure that the algorithm will be part of the strategic priorities of the department (especially in settings where the technical resources cannot scale to tackle multiple projects). The division can help ensure that the algorithm is implemented in a way that answers the clinical question, is acceptable by the division members, and is safe for patients in context of the domain expertise. In addition, for the algorithm to succeed, the department needs to allocate resources to assist with education, algorithm validation, and practice alignment.

A key consideration for these AI algorithms is to extend its value beyond the radiologists to clinicians who might incorporate the AI output as part of their patient care management. For these clinicians, it is imperative that the ordering mechanism for these algorithms is incorporated in their workflow. The wording of the order needs to be approved by the stakeholder, and the order needs to be available in the proper clinical context. Thus, it is critical that systems beyond the ones used by the radiologist are prepared for interoperability and enabled to receive the findings of the AI algorithm. This will support effective delivery of AI output to the designated clinician at the point of care (Figure 1).

#### Stakeholder Engagement

An essential consideration for this operational framework is to address the needs of the diverse group of stakeholders who will be interested, involved or impacted by AI in the clinical practice.^12^ Table provides a summary of the various types of stakeholders, their role within the organization, and their key attributes that need to be considered as part of the operational framework.

**Table:** Various Types of Stakeholders and Their Role Within the Organization

Type of Stakeholder	Organizational Role	Key Roles
Patient		○ Understand the AI output and impact on patient management ○ Direct use ○ Express considerations ○ Participate in report output format
Innovator	○ Physicians ○ Investigators ○ Employee	○ Identify problem ○ Domain expertise ○ Bigger picture view ○ Workflow understanding ○ Experience with AI
Translator	○ Data scientists ○ IT experts ○ Engineers ○ Business analyst ○ Education specialist ○ Study coordinators	○ Stakeholder engagement and management ○ Technical expertise ○ Drive collaboration ○ Manage communication and multi-party expectations ○ User research and testing
Adopter	○ Physicians ○ Providers ○ Patients ○ Ancillary Staff	○ Define workflow ○ Set expectations ○ Provide feedback for product optimization
Leader	○ Physicians ○ Department Leadership ○ Governance groups ○ Institutional Leadership	○ Support AI efforts ○ Strategic goals and business cases ○ Overall vision
Adherence representatives	○ Quality assurance specialist ○ Regulatory affairs specialist ○ Risk management expert	○ Quality management system ○ Regulatory requirements ○ International standards

Abbreviation: AI, artificial intelligence.

Innovators are vital for identifying and solving problems with AI, necessitating adaptable operational structures to support their diverse needs. From novice to expert, these individuals require guidance and resources to develop AI solutions, ensuring the realization of its full potential across various domains.

Translators play a crucial role in converting innovators’ AI concepts into practical applications for clinical settings. These experts, such as medical physicists and data scientists, require training and engagement to seamlessly integrate AI into health care environments. Similarly, adopters, such as physicians,^13^ determine the efficacy of AI in patient care, necessitating clear mechanisms for their involvement and feedback. Meanwhile, leaders within organizations shape the vision for AI implementation, fostering innovation and providing necessary resources for teams to thrive and evolve.

#### Change Management

The AI is a disruptive technology poised to transform care delivery. Many users are cautious about the impact of that disruption and its implications on their roles within the organization. The AI algorithms that meet the definition of a medical device have the additional requirement of formalizing the change management process so it complies with FDA regulations and international standards. The level of change management and the oversight of the process are proportional to the risk of the device (ie, higher risk devices require additional management and FDA oversight of those activities). Health care institutions have the unique challenge of developing these processes in a manner that meets the industry standards expected by regulators. Medical device companies already have the infrastructure in place to conduct these activities. A comprehensive change management plan will need to be outlined, and best practices of change management will need to be implemented before deployment of the AI algorithms into the clinical practice. The change management plan must address stakeholder engagement, structured training programs, transparent communication, and ongoing monitoring. Early and active engagement of key stakeholders, such as clinicians and administrative staff, is vital to garner support and mitigate resistance. Tailored training sessions should be designed to equip health care professionals with the necessary skills and knowledge to effectively use AI tools, whereas clear communication channels ensure all parties are informed about the implementation process and any changes to workflows.

#### Talent

Sourcing talent for the translation of AI algorithms although maintaining the quality management system (QMS) or integrating in the clinical workflow in the field of radiology is quite challenging. To build a successful team, domain expertise is required. Understanding the tool set utilizing the clinical workflow presents unique challenges. In cases where onboarding experienced personnel is not feasible, creation of educational material, such as work instructions capturing the know-how is crucial to enable smaller teams to follow the same pathway.

#### Education and Training

Role-based training and education are critical components for the successful implementation of AI algorithms in clinical workflows. Various roles, such as ordering providers, radiologists, IT personnel, data scientists, education specialists, supervisors, and staff such as technologists and image analysts, must be equipped with both specialized knowledge in their respective areas and a comprehensive understanding of the entire AI process. Training plans tailored to each role should address core competencies, learning objectives, practice gaps, and preferred learning methods.

Education efforts should target specific roles, such as providing training for referring clinicians and radiologists on algorithm ordering and result interpretation, equipping IT scientists with necessary software development skills, and instructing laboratory aides and personnel on segmentation software usage. The QMS training is essential, particularly for employees involved in design control and risk management processes, ensuring alignment to FDA consensus standards such as ISO 13485, QMS, ISO 14971, application of risk management, and IEC 62304, and software lifecycle process. Onboarding processes should include formal training sessions, and comprehensive work instructions and reference materials should be readily accessible to support ongoing learning and project execution.

### Integration

Successful adoption of AI algorithms in routine clinical practice is contingent on seamless integration of these AI algorithms in the clinical workflow in a way that does not deviate from the product’s intended use and with little or no disruption of existing workflows. A concerted amount of time needs to be invested in mapping the current state workflows and how the care team operates as part of that workflow. There will be many workflows that need to be mapped, such as downtime workflows, system failure workflows, etc. A nuanced mapping of the clinical workflow can provide a comprehensive understanding of the system and how the users operate within that system. Product teams should account for sufficient time and resource allocation to conduct usability and human factors testing.

Seamless integration is key to help establish a minimally disruptive future-state workflow that includes the integration of the AI algorithm in the clinical practice. The more integrated the AI algorithm is within the pre-established clinical workflows, the higher the probability for its successful adoption in the clinical practice. An example workflow for an orderable AI is provided in Figure 2. On contrary, AI algorithms that do not consider pre-existing workflows have a much higher chance of not being used.

**Figure 2:**
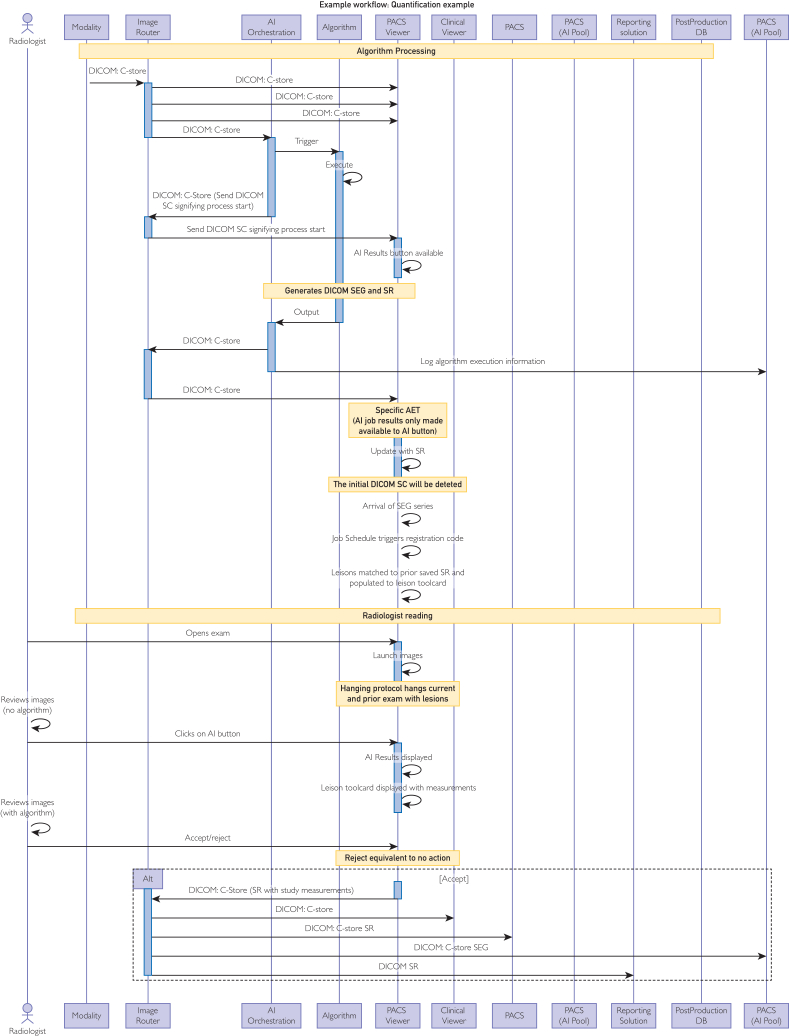
Example of the clinical workflow of a manually invoked quantification algorithm. The clinician places the order, the images are acquired, and the AI is applied in the background when an image series that match some prespecified criteria is detected. The radiologist reviews the AI output. If the AI output is not correct, it is manually corrected or routed to the 3D laboratory for corrections. Once the output is corrected, the radiologist can incorporate the volumetric measurements to his report. AI, artificial intelligence.

Because of the explosion of AI solutions, one thing to consider is how results from multiple AI algorithms are presented to the radiologist. If the integration mechanisms are considerably different, this has the potential to lead to confusion and reluctance in utilizing the algorithms. One possible solution to this problem is an AI dashboard that summarizes findings and allows the radiologist to select the appropriate analytic to display.

In addition, the way AI algorithms are triggered can pose additional challenges. The following example may illustrate this challenge. Take the example of a patient visiting the radiology department for a computed tomography scan for lung cancer screening, where an always on AI algorithm calculates a coronary calcium score of 300. Creating a workflow that will ensure that these patients get appropriate treatment on the basis of this finding can be challenging. Was the AI output correct? Was the patient already treated? If no, how we can notify the primary physician outside of the institution that the computed tomography scan was performed? An additional question is whether or not patients should be notified of the result, and, if so, when?

The AI algorithms, particularly those capable of identifying incidental findings, introduce significant complexity into clinical workflows. These algorithms, designed to detect abnormalities outside the primary scope of investigation, necessitate robust mechanisms for communication and follow up within the health care system. The challenge lies in ensuring that these incidental findings are accurately communicated to the relevant health care providers, such as radiologists, primary care physicians, and specialists, in a timely and efficient manner. This requires a seamless integration of AI outputs into EHRs and notification systems, enabling immediate attention and appropriate clinical action. In addition, there must be clear protocols in place to determine the urgency and relevance of these findings, considering patient history and clinical context, to avoid unnecessary duplicative work, patient anxiety, or procedures. The ability to effectively manage and communicate incidental findings is critical, as it can significantly impact patient care pathways, necessitating a multidisciplinary approach to ensure that such information leads to beneficial clinical outcomes without overwhelming the system with false positives or clinically insignificant alerts.

### Regulatory

#### QMS and FDA Clearance

The FDA has cleared nearly 700^14^ AI-enabled medical devices and has made significant efforts to accommodate and provide guidance for developing and deploying AI into clinical practice.^15^ The FDA quality systems regulations may be applicable when AI functionality is intended for use in the cure, mitigation, treatment, prevention, or diagnosis of a disease or condition and does not fall under an exempt software category. The FDA’s digital health policy navigator provides a useful tool to help determine whether or not a digital health solution is regulated by the FDA.^16^ However, due to heightened risk and the image-based nature of radiology, many radiology functions fall under FDA’s jurisdiction, which is evident in the fact that over 75% of the FDA-cleared AI-based medical devices are in radiology.^14^

Quality systems regulation applies to both commercial AI and in-house AI solutions developed at a health care organization. In addition to representing state-of-the-art best practices, FDA-recognized standards, such as ISO 14971, application of risk management, and IEC 62304, software lifecycle process, help guide the rigor and evidence generated to ensure meeting standards and requirements for safety, efficacy, and equity. As with any other medical device, the applicable requirements are determined on the basis of the AI algorithm’s intended use(s), end user(s), and risk profile. The FDA also continues to release updated guidance on their expectations for adherence to good ML practices (GMLP),^17^ which can be baked into existing standards and processes. Collectively, the standards and processes followed make up the QMS.

An FDA-compliant QMS combined with GMLP facilitates the development, deployment, and maintenance of high-quality, validated AI algorithms that contain appropriate risk-mitigations to minimize harm to the end user and feedback loops and oversight to create accountability and identify areas for improvement. A QMS can also establish credibility and trust within a provider organization that is deploying an AI algorithm in the clinical practice. In addition, an FDA-compliant QMS utilizes the most current standards and decades of regulatory science to aid in standardization of terms and ensure state-of-the-art manufacturing principles and safety practices are accounted for. Built within a health care organization, an FDA-aligned QMS creates a standard language, predictable roadmaps, and the assurance that the organization and each individual product is managed and controlled in a safe, effective, and ethical manner. Finally, a QMS is necessary for FDA clearance of AI-based medical devices, which can in turn expand opportunities for partnership or commercialization of health system-developed AI solutions.

The FDA is exploring frameworks to better accommodate the more iterative nature of software, including the concept of an algorithm change protocol with prespecified acceptance criteria that the FDA could review for safety and efficacy at the time of clearance/approval, which would allow for a defined range of software changes that do not require FDA review.^17^

#### Validation Studies

Before deploying an AI algorithm, the product team must complete a formal validation study under the oversight of an institutional review board. The study is one of the final steps in collecting evidence that user needs are met, risks are mitigated to an acceptable level, and the solution works in its clinical setting as intended. Validation requirements are established as a key component to an organization’s QMS. Furthermore, to enable commercialization through an FDA premarket review and clearance, validation studies will likely require multisite validation. In some cases, multisite validation can be sufficiently conducted using retrospective datasets, but in other cases, prospective validation is necessary to ensure safety, efficacy, and equity, for example, in cases where user operability plays an important role in mitigating risks.

## Discussion

This paper sheds light on the multifaceted challenges encountered in developing and integrating in-house AI algorithms within radiology workflows, particularly as organizations transition from research to clinical deployment. This paper focuses on challenges following after a generalizable algorithm has been developed and validated. These challenges span operational, technical, clinical, and regulatory domains, necessitating strategic goal-setting and support mechanisms to navigate successfully. Identifying these challenges upfront is crucial for defining the scope and addressing gaps in the translation process.

The complexity of developing comprehensive solutions is magnified by the diversity of tools and systems in organizations seeking AI integration, varying technical capabilities, and operational scales. A pragmatic approach involves starting on a small scale, educating organizational members about AI integration, and considering co-development opportunities to lower entry barriers, though challenges in intellectual property and data sharing agreements may arise. Moreover, educational initiatives and communication channels are essential to inform radiologists about algorithm failures and integrate this knowledge into daily practice, alongside quantitative analyses to evaluate AI’s impact on diagnostic accuracy, efficiency, and patient outcomes.

The successful adoption of AI in medical imaging hinges on robust algorithms and efficient workflows. Radiologists face challenges when AI algorithms fail or produce poor-quality output, straining workload, and financial sustainability. Thus, ensuring reliable algorithms and streamlined workflows is crucial for realizing AI’s potential in health care.

The AI integration in radiology complicates workflows, necessitating effective management and communication of incidental findings through seamless integration into EHRs and clear follow-up protocols. Multidisciplinary teams trained on effective data management and algorithm standards can lead to efficient implementation and monitoring that addresses biases inherent to AI and ensures a robust change management process is in place to detect and mitigate the risk of patient harm resulting from errors or external factors impacting algorithm performance. Collaboration among domain experts is essential for addressing financial challenges and ensuring the sustainable benefits of AI technologies in health care. Finally, although this paper focuses on radiology, the discussed challenges are relevant to various specialties, highlighting common hurdles in different clinical settings.

The aforementioned challenges go beyond the well-documented issues of algorithm training, bias, and interpretability.^18^^,^^19^

## Conclusion

In conclusion, the integration of AI-enabled algorithms into the radiology workflow presents a multifaceted challenge spanning operational, technical, clinical, and regulatory domains. Overcoming these hurdles necessitates strategic planning, educational initiatives, and careful consideration of the implications for radiologists’ workloads. Institutions must navigate these challenges although understanding the benefits and limitations of both approved and in-house-developed AI tools. Collaboration among diverse stakeholders is essential to ensure that AI technologies enhance patient care without adding unnecessary burdens. As health care evolves with AI integration, maintaining a focus on patient safety, efficacy, and overall clinical outcomes is paramount.

## Potential Competing Interest

The authors report no competing interests.
